# Failure Prediction of Notched Composites Using Multiscale Approach

**DOI:** 10.3390/polym14122481

**Published:** 2022-06-18

**Authors:** Young W. Kwon

**Affiliations:** Department of Mechanical & Aerospace, Engineering, Naval Postgraduate School, Monterey, CA 93943, USA; ywkwonl@nps.edu

**Keywords:** failure criteria, multiscale, composites, notch

## Abstract

This paper presents multiscale-based failure criteria to predict the failure of polymer composites with any shape of defect. The multiscale technique consists of bi-directional processes called upscaling and downscaling processes to link two different length scales. One is the microscale at the fiber and matrix material level, and the other is the macroscale at the homogenized composite material level. Failure criteria are applied to the microscale level such as micro-stresses and/or micro-strains occurring at the fiber and matrix material level. Recently proposed unified failure criteria are applied to the micro-stresses and/or micro-strains to predict failure at a notch. The new failure criteria have two parts, and they can be applied to any shape of defect. One is the stress or strain condition, and the other is the stress or strain gradient condition. For failure to occur, both conditions must be satisfied simultaneously. The failure criteria provide not only failure locations but also directions of failure propagation.

## 1. Introduction

Developing reliable failure criteria for any material is very important for the proper design of load-carrying structural members to avoid failure. As a result, such effort has been undertaken very extensively, especially for non-isotropic materials such as fibrous composites because they have different strengths depending on the loading direction. Fibrous composite materials have multiple failure modes. If a composite structure has laminated layers, failure may also occur between layers, so-called interlaminar failure.

Some failure criteria for fibrous composite materials were expressed as a single equation such as Tsai–Hill and Tsai–Wu criteria [1,2,3]. These failure criteria include multiple failure strengths and failure modes in one equation. These criteria are easy to apply but they do not clearly provide what failure mode causes the failure under combined loading. Others proposed multi-part failure criteria depending on failure modes. There are multiple equations for one set of failure criteria. Those examples include the criteria proposed by Hashin and his group [4,5]. A review and classification of failure criteria for fibrous composite materials were published in Ref. [6]. Various composite failure criteria were explored and compared through the Worldwide Failure Exercise [7].

Almost all of those failure criteria, to the best knowledge of the author, used the stresses and strains occurring at the lamina level. In other words, composite material level stresses and/or strains were used in the failure criteria, which are called macro-stresses and macro-strains hereafter. Those macro-stresses/strains are easy to compute using finite element analyses of composite structures. However, the macro-stresses/strains are mathematically homogenized values of those occurring in the constituent materials such as the fiber and matrix materials. As a result, the macro-stresses/strains do not represent the physical quantity experienced by the fiber and matrix materials. The stresses and strains carried by the fiber and binding matrix materials are called micro-stresses and micro-strains.

A more recently proposed set of failure criteria used the micro-stresses/strains instead of macro-stresses/strains [8,9,10]. Therefore, more physical stresses and strains were considered for the failure criteria. In order to use micro-stresses/strains for the failure criteria, a multiscale approach was adopted such that micro-stresses/strains could be determined from the macro-stresses/strains. Because the new failure criteria were applied to the fiber and matrix material level, the failure modes were fiber failure, matrix failure, and fiber–matrix interface failure. For example, fiber splitting, transverse matrix cracking, and interlamination failure are either matrix failure, fiber/matrix interface failure, or some combination of them.

All the failure criteria mentioned above are not applicable to predict failure of structural members with a crack or a hole. For example, the stress field is singular at the crack tip, which makes the previous failure criteria not applicable for a structural member with a crack. To overcome this, fracture mechanics was used for a structural member with a crack.

A structural member with a hole does not have a stress singularity but a stress concentration. Failure loads predicted by using the previous failure criteria do not agree well with experimentally measured failure loads. Furthermore, fracture mechanics cannot be used for a hole unless an assumed crack is considered at the edge of the hole. To this end, other failure criteria were proposed to predict the failure loading of a structural part with a hole [11,12,13,14,15,16,17,18]. In summary, three different kinds of failure criteria have been used to predict failure loading depending on whether a structural member has a defect or not, and what kind of defect it is like a crack or a cutout.

Very recently, the author proposed a unified failure criterion for brittle materials such as polymer composites [19,20]. The unified criterion can be applied to any structural member regardless of whether it has a crack, a cutout, or none of them. The unified failure criteria have two parts. In order for failure to occur, both parts must be satisfied simultaneously. The first part is the stress-based criterion, and the second part is the stress gradient-based criterion.

In this study, the unified failure criteria were coupled with the multiscale approach as discussed above such that the unified failure criteria could be applied in terms of the micro-stresses and micro-strains to predict failure of fibrous composite specimens regardless of whether they have any kind of defect or not. The next section presents the multiscale approach, which is followed by the unified failure criteria in terms of the micro-stresses and micro-strains. Then, some examples are provided to validate the proposed multiscale-based unified failure criteria. Finally, conclusions are given.

## 2. Multiscale Analysis

**Figure 1:**
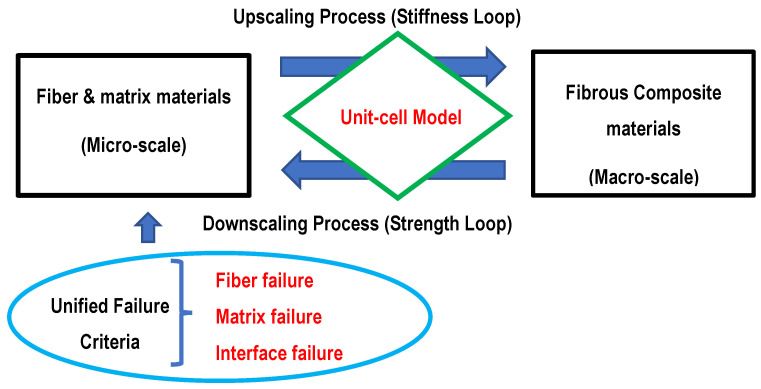
Multiscale analysis of macro- and micro-scales.

The multiscale analysis used in this study has bidirectional processes between two different length scales. One of them is the macro-scale in terms of composite materials, and the other is the micro-scale in terms of the fiber and matrix materials, as sketched in Figure 1. The multiscale analysis requires continuous iterations of the bidirectional processes that consist of upscaling and downscaling processes.

The upscaling process is the stiffness loop because the process computes the effective material properties of fibrous composite materials from the properties of the fiber and matrix materials and their volume fractions, as expressed below:

(1) $${\bar{E}}_{ijkl}=f_{1}(E_{ijkl}^{f},E_{ijkl}^{m},v^{f})$$

where ${\bar{E}}_{ijkl}$ is the homogenized material modulus of a fibrous composite, $E_{ijkl}^{f}$ and $E_{ijkl}^{m}$ are the elastic moduli of the fiber and matrix materials, respectively, and $v^{f}$ is the fiber volume fraction. In this model, it is assumed that $v^{f}+v^{m}=1$, where $v^{m}$ is the matrix volume fraction. That is, no void is assumed. However, the current model can be easily modified for any potential void. The details of the function in Equation (1) are discussed later. Initially, the virgin material properties are used for the fiber and matrix materials. As damage and failure occur, degraded material properties are used.

The downscaling process is the strength loop that computes the micro-stresses/strains from macro-stresses/strains. First, micro-scale strains are computed from the macro-strains as follows:

(2) $$\varepsilon_{ij}^{f,m}=f_{2}(E_{ijkl}^{f},E_{ijkl}^{m},v^{f},{\bar{\varepsilon }}_{ij})$$

where $\varepsilon_{ij}^{f,m}$ is the micro-strains of the fiber and matrix materials, and ${\bar{\varepsilon }}_{ij}$ is the macro-strains of the composite material. Then, micro-stresses are determined from the micro-strains as follows:

(3) $$\sigma_{ij}^{f}=E_{ijkl}^{f}\varepsilon_{ij}^{f}$$

(4) $$\sigma_{ij}^{m}=E_{ijkl}^{m}\varepsilon_{ij}^{m}$$

in which $\sigma_{ij}^{f}$ and $\sigma_{ij}^{m}$ are the micro-stresses of the fiber and matrix materials, respectively.

Both upscaling and downscaling processes are repeated as the applied load increases or damage and failure evolve. As the finite element method is used to analyze composite structural members, the upscaling and downscaling processes are applied to every finite element, especially at their numerical integration points. Therefore, the computational efficiency of the multiscale analysis is directly dependent on the efficiency of the upscaling and downscaling processes.

Analytical forms of solutions are developed to make the multiscale analysis computationally efficient. In other words, Equations (1) and (2) are expressed analytically such that any additional numerical modeling and analysis are not needed. To this end, a unit-cell model is used for the present multiscale analysis, which is presented below.

**Figure 2:**
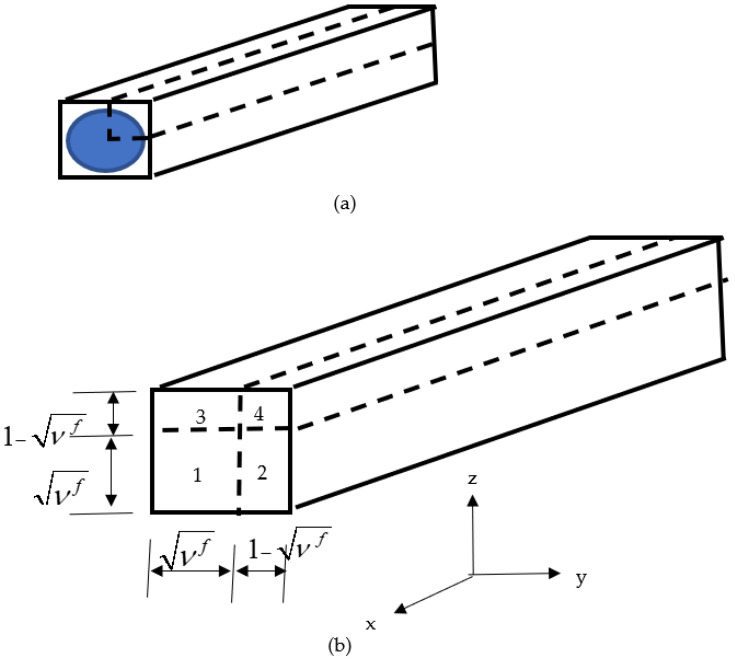
Unit-cell model: (**a**) full geometry and (**b**) quarter model (the *x*-axis is the fiber orientation, and $\nu^{f}$ denotes the fiber volume fraction).

A unit-cell is as sketched in Figure 2a. Because of the quarter symmetry, only one quarter of the unit-cell is considered, and the fiber geometry is simplified as shown in Figure 2b. In this unit-cell model, the fiber denoted by sub-cell #1 in Figure 2b does not necessarily represent a single fiber. Instead, it is the representation of many fibers around a numerical integration point of every finite element. Therefore, the actual shape of fibers is not important. Instead, the collective behavior of those many fibers is considered in the model. In other words, the fiber sub-cell in the unit-cell model represents the average fiber behavior of the fibers inside a small section that is around each numerical integration point of finite elements. Because of this, the fiber cross-section is simplified as a square shape and whose size is equal to $\sqrt{\nu^{f}}$ because the fiber volume fraction is $v^{f}$.

The quarter of the unit-cell has four sub-cells as sketched in Figure 2b. One of the sub-cells represents the fiber material and the remaining sub-cells are for the matrix material. For mathematical simplicity, every sub-cell is assumed to have uniform stresses and strains, which are denoted as $\sigma_{ij}^{n}$ and $\varepsilon_{ij}^{n}$, respectively, where the superscript *n* indicates the sub-cell number. This means that $\sigma_{ij}^{1}$ and $\varepsilon_{ij}^{1}$ are the micro-stresses and micro-strains of the fiber, respectively, while others are for the matrix. The coordinate system is set such that the *x*-axis is along the fiber orientation.

The expressions as stated in Equations (1) and (2) are derived as below. First, equations of equilibrium among micro-stresses are applied to every interface of neighboring sub-cells. For example, between sub-cells #1 and #2, we have

(5) $$\sigma_{yy}^{1}=\sigma_{yy}^{2},\;\sigma_{xy}^{1}=\sigma_{xy}^{2},\;\sigma_{yz}^{1}=\sigma_{yz}^{2}$$

These kinds of equations of equilibrium are applied to every interface.

Micro-strains of sub-cells also need to maintain compatibility of deformation. For instance, along the *x*-axis, sub-cell micro-strains have the following compatibility.

(6) $$\varepsilon_{xx}^{1}=\varepsilon_{xx}^{2}=\varepsilon_{xx}^{3}=\varepsilon_{xx}^{4}$$

Along the *y*-axis, the compatibility of micro-strains is

(7) $$\sqrt{\nu^{f}}\varepsilon_{yy}^{1}+\left(1-\sqrt{\nu^{f}}\right)\varepsilon_{yy}^{2}=\sqrt{\nu^{f}}\varepsilon_{yy}^{3}+\left(1-\sqrt{\nu^{f}}\right)\varepsilon_{yy}^{4}$$

Similar compatibility equations among micro-strains can be written.

The constitutive equations for each sub-cell are given in Equations (3) and (4) if superscript *f* is replaced 1, and superscript *m* is replaced by 2, 3, or 3. The composite level macro-stresses and macro-strains also have their constitutive equation, as expressed below:

(8) $${\bar{\sigma }}_{ij}={\bar{E}}_{ijkl}{\bar{\varepsilon }}_{kl}$$

where the overbar indicates that the quantities are at the composite level. Finally, the macro-stresses and macro-strains are assumed to be the volume average of micro-stresses and micro-strains, respectively, as expressed below:

(9) $${\bar{\sigma }}_{ij}=\nu^{f}\sigma_{ij}^{1}+\sqrt{\nu^{f}}\left(1-\sqrt{\nu^{f}}\right)\left(\sigma_{ij}^{2}+\sigma_{ij}^{3}\right)+{\left(1-\sqrt{\nu^{f}}\right)}^{2}\sigma_{ij}^{4}$$

(10) $${\bar{\varepsilon }}_{ij}=\nu^{f}\varepsilon_{ij}^{1}+\sqrt{\nu^{f}}\left(1-\sqrt{\nu^{f}}\right)\left(\varepsilon_{ij}^{2}+\varepsilon_{ij}^{3}\right)+{\left(1-\sqrt{\nu^{f}}\right)}^{2}\varepsilon_{ij}^{4}$$

Solving all the equations mentioned above eventually results in the final expressions as given in Equations (1) and (2). The detailed derivation was given in Refs. [8,21] and is omitted here to save space. At the end, no additional numerical model is used for the multiscale analysis, to make it computationally efficient.

## 3. Failure Criteria

Before presenting specific failure criteria for fibrous composite materials, a recently proposed unified failure criterion is discussed for brittle materials including polymer composites. The unified failure criterion has two parts. For failure to occur at a given location of a structural member, both parts of the criterion must be satisfied simultaneously. If only one part of the two is satisfied, failure does not occur [19,20].

The first part of the failure criterion is the stress-based failure criterion. This part states that the effective stress of the applied load should be at least equal to or greater than the failure strength of the material. The failure strength is obtained using a typical tensile test coupon such as dog-bone-shape specimens or rectangular specimens with end tabs. The first part of the failure criterion is expressed mathematically as

(11) $$\sigma_{eff}\geq \sigma_{fail}$$

where $\sigma_{eff}$ is the effective stress that may be different for different materials, and $\sigma_{fail}$ is the failure strength of the material. Some materials have different strengths between tension and compression. In that case, proper strength is used depending on the loading direction. To apply to both tension and compression, $\sigma_{eff}$ and $\sigma_{fail}$ are the absolute values. This criterion checks potential locations for failure depending on the stress level of those spots.

The second part of the failure criterion is the stress-gradient-based failure criterion. This part checks the neighboring state for failure because the stress gradient is a value depending on the stress variation from the present location to an immediate neighbor. The second part of the failure criterion is expressed as

(12) $$\sigma_{eff}^{3}{\left|\frac{d\sigma_{eff}}{ds}\right|}^{-1}\geq 2E_{eff}\kappa_{fail}=2{\bar{\kappa }}_{fail}$$

in which *s* is along the failure direction, $E_{eff}$ is the effective elastic modulus, and $\kappa_{fail}$ is another material constant for failure. As the singular crack tip stress solution is substituted into Equation (12) from the linear fracture mechanics, it shows that $\kappa_{fail}$ is directly related to fracture toughness or critical energy release rate as follows:

(13) $$\kappa_{fail}=\frac{K_{Ic}^{2}}{2\pi E}=\frac{G_{c}}{2\pi }$$

for the mode I fracture of an isotropic material under the plain stress condition, where $K_{Ic}$ is the fracture toughness of the first mode, $G_{c}$ is the critical energy release rate, and E is the elastic modulus. The failure direction (or failure path) may be known or unknown a priori. For example, if the loading, geometric, and boundary conditions are all symmetric, failure is expected along the symmetric axis. If this is not the case, the failure direction may not be known a priori. In this situation, the potential failure direction is determined in the direction that results in the minimum value of the stress gradient in terms of its magnitude.

Structural members or test specimens may have one of the three possible geometric configurations. The first one has neither a crack nor a cutout. The second one has a crack. The third one has a cutout such as a circular hole. We present subsequently how the unified failure criteria are applied to the three cases.

As the first case, we consider a tensile coupon that provides the tensile strength of the material under test. In this case, stress is unform across the width of the test coupon as it is under uniaxial loading, which means the stress gradient is zero in terms of the stress variation along the width of the specimen. In this situation, the second part of the failure criterion, i.e., Equation (12), is explicitly satisfied regardless of the magnitude of the effective stress. Hence, the first part determines the failure of the specimen. That is why we obtain the failure strength of the material from the tensile test coupon.

The second case has a crack, which gives a stress singularity if the material is elastic. That is, the stress becomes infinity at the crack tip for any applied load other than zero magnitude. This indicates that the first part of the failure criterion is explicitly satisfied at the crack tip. Therefore, the second part determines failure loading, and fracture toughness and/or critical energy release rate can be used to obtain the failure load.

A plate with a circular hole is considered as the third case, for which none of the two parts are explicitly satisfied. As a result, both parts of the criterion must be tested to check for potential failure. However, for a reasonable size of a hole that is neither too large nor too small, the second part of the criterion determines the failure load. In other words, when the stress at the edge of a hole reaches the failure strength of the material, failure does not necessarily occur for a reasonable size of a hole. The failure load computed using the second part of the failure criterion yields a stress at the edge of the hole greater than the failure strength when failure occurs at the edge of the hole.

The unified failure criterion may also be expressed in terms of the effective strain $\varepsilon_{eff}$ instead of the effective stress $\sigma_{eff}$. The unified failure criterion is applied to the multiscale analysis of fibrous composites, the failure of which is determined based on the failure of constituent materials such as the fiber and matrix. As a result, failure is assessed in terms of the micro-stresses and/or micro-strains [9]. There are three failure modes at the constituent material level: fiber failure, matrix failure, and fiber–matrix interface failure. As a result, there is one failure criterion for each failure mode, resulting in three failure criteria all together.

Fibers are the major load-carrying elements in the composite, and fiber failure is the most critical for fibrous composites. Fibers support the applied load mostly along the longitudinal direction of the fibers. Transverse loading to fibers can also be supported by the fibers. However, matrix materials embedded with fibers will fail before fibers fail under the transverse loading, which eventually yields failure of the composite. Hence, fiber failure is considered in terms of the loading along the fiber direction. The fiber failure criterion also has two parts as discussed previously for the unified failure criterion. The failure criterion for fibers uses the following effective stress in association with Equations (11) and (12).

(14) $$\sigma_{eff}^{f}=\sqrt{{\left(\sigma_{x}^{f}\right)}^{2}+\left(\frac{E_{x}^{f}}{G_{xy}^{f}}\right)\left[{\left(\sigma_{xy}^{f}\right)}^{2}+{\left(\sigma_{xz}^{f}\right)}^{2}\right]}$$

where superscript *f* denotes the fiber, subscript *x* is along the fiber orientation, and *E* and *G* are elastic and shear moduli, respectively. The fiber effective stress must satisfy both parts of Equations (11) and (12) for fiber failure to occur. Fibrous composites have, in general, different strengths between tension and compression because of fiber buckling. Thus, different strength values should be considered depending on the loading direction.

The matrix materials used in polymer composites mostly behave brittle, and the materials may be assumed isotropic. In this case, the effective stress used for matrix failure is the maximum or minimal normal stress in the matrix material depending on whether the material is under tension or compression. That is,

(15) $$\sigma_{eff}^{m}=\{\begin{array}{cc} \sigma_{\max}^{m} & \mathrm{if}\;\mathrm{tension}\\ \sigma_{\min}^{m} & \mathrm{if}\;\mathrm{compression} \end{array}$$

where superscript *m* denotes the matrix material.

Interface failure between fiber and matrix materials is the last failure mode. The failure criterion for fiber–matrix debonding is more complex than the previous failure criteria. Peeling (or tensile) normal stress contributes to the interface failure, but compressive normal stress has a negligible contribution to the interface failure. Under that condition, the criterion for the fiber–matrix interface failure on the *xz*-plane is expressed as

(16) $${\left(\frac{\sigma_{xy}^{m}+\sqrt{v^{f}}\left(\sigma_{y}^{m}-\sigma_{x}^{m}\right)}{\tau_{fail}^{int}}\right)}^{2}+{\langle \frac{\sigma_{y}^{m}}{\sigma_{fail}^{int}}\rangle }^{2}\geq 1$$

in which $\tau_{fail}^{int}$ and $\sigma_{fail}^{int}$ are the failure strengths of the interface under shear and normal stress only, respectively. In addition, $\langle \sigma \rangle =\frac{\sigma +\left|\sigma \right|}{2}$ is the Macaulay operator, which states that only tensile normal stress contributes to the interface failure. The same kind of failure criterion can be written for the *xy*-plane interface failure if the *y*-component is replaced by the *z*-component.

## 4. Description of Specimens and Experiment

Laminated carbon fiber composite specimens had a circular hole at the center. The nominal specimen size was 140 mm long, 24 mm wide, and 1.7 mm thick. The distance between two grips of the uniaxial testing machine was 100 mm. The specimens had a hole of a different diameter. There were two different layups. One layup had a cross-ply orientation such as [90°/90°/0°/0°/90°/90°]_s_, and these specimens were called Cross-Ply (CP) specimens, which were loaded along the 0° orientation. The other laminated composite had a layup of [0°/0°/+45°/−45°/90°/90°]_s_. The layup was not exactly quasi-isotropic, because the numbers of +45° and −45° layers were not equal to those of 0° and 90° layers. However, the specimens were called the Quasi-Isotropic (QI) specimens for simplicity. Loading for QI specimens was also applied in the 0° axis.

First, all the perforated composite specimens were tested under uniaxial tensile loading at the speed of 0.03 mm/s using an Instron testing machine. Three to five samples were tested for the same type of specimen, i.e., the same size of hole and the same layup orientation, in order to check for any statistical variation in the test results. In addition, CP and QI specimens as well as unidirectional specimens made of the same fiber and matrix materials were tested without perforation to determine their strength and stiffness.

## 5. Results and Discussion

The experiment of the unidirectional specimens gave the elastic moduli along the longitudinal and transverse directions of 115 GPa and 7.71 GPa, respectively. The stress–strain curves were very linear up to failure. The tensile failure strength of the unidirectional composite was 1350 MPa and 52 MPa in the longitudinal and transverse directions, respectively. The upscaling process of the multiscale analysis resulted in the effective moduli of the unidirectional composite as follows using the material properties given in Table 1, which lists the fiber and matrix material properties. Most of the material properties in Table 1 were obtained from the material manufacturers’ data, while others were obtained at the lab. The predicted longitudinal elastic modulus was 112 GPa, and the transverse elastic modulus was 7.61 GPa. Thus, the predicted stiffness of the unidirectional composites was very close to the measured stiffness. The difference was less than 3%. The fiber and matrix material properties were used for the multiscale analysis in this study.

To predict failure of the perforated composite specimens, we need both failure values for Equations (11) and (12). The failure strength for Equation (11) can be determined from the fiber and matrix strength. The experimental failure strength of the unidirectional composite was 1380 MPa and 51.4 MPa along the longitudinal and transverse directions, respectively, while the predicted strength was 1310 MPa and 50.4 MPa. The comparison of strength in the longitudinal and transverse directions of the unidirectional composites was excellent between the experimental and numerical results.

However, the failure value ${\bar{\kappa }}_{fail}$ in Equation (12) could not be obtained from the specimens without any cutout, because the typical tensile test coupon had zero stress gradient across the width of the specimen. Therefore, the failure value ${\bar{\kappa }}_{fail}$ was obtained using one of the perforated composite specimens. To this end, one of the specimens with a circular hole was selected to determine the failure value. The CP specimens had a hole diameter of 3 mm or 6 mm, while QI specimens had a hole diameter of 6 mm or 8 mm. Among those, the CP specimen with a 6 mm hole was used to determine ${\bar{\kappa }}_{fail}$. This was an arbitrary choice. Once the ${\bar{\kappa }}_{fail}$ value was determined from one specimen, the same ${\bar{\kappa }}_{fail}$ value was used for the rest of specimens to predict the failure load regardless of the lamination angle. That is an advantage of the multiscale approach. Because failure is determined using the micro-stresses/strains, the macroscale strength of the laminated composite is not required, which varies depending on the layup angles. The CP and QI specimens had different strengths and stiffnesses at the macroscale. The lamination theory can be used to determine the stiffness of a laminated composite as a function of layup angles. However, the strength of a laminated composite is not easy to obtain. Mostly, additional tests are required to obtain the strength of a new laminated composite.

Before predicting the failure load, the finite element mesh was examined to make sure it was acceptable in terms of accuracy. As the failure criterion uses both stress and stress-gradient values, their finite element solutions were compared to the available analytical solution for an infinite plate with a circular hole at the center under uniaxial loading. The normal stress $\sigma_{y}$ at the edge of a hole is expressed along the *x*-axis as follows if the uniaxial loading $\sigma_{o}$ is applied in the *y*-axis direction:

(17) $$\sigma_{y}=\frac{\sigma_{o}}{2}\left[\left(1+\frac{a^{2}}{x^{2}}\right)+\left(1+\frac{3a^{4}}{x^{4}}\right)\right]$$

where *a* is the hole radius and *x* is measured from the center of the hole. The stress concentration factor at the edge of the hole is 3 at *x* = *a*. The stress gradient at the edge of the hole is computed by taking the derivative of Equation (17) with respect to *x*, which results in

(18) $$\left|\frac{d\sigma_{y}}{dx}\right|=\sigma_{o}\left(\frac{a^{2}}{x^{3}}+\frac{6a^{4}}{x^{5}}\right)$$

The evaluation of Equation (18) at *x* = *a* yields

(19) $$\left|\frac{d\sigma_{y}(a)}{dx}\right|=\frac{7\sigma_{o}}{a}$$

That is, the stress gradient for an applied unit stress is inversely proportional to the hole radius *a*. If *a* is 2 mm and $\sigma_{o}=1$ Pa, Equation (19) gives the stress gradient of 3500 Pa/m.

**Figure 3:**
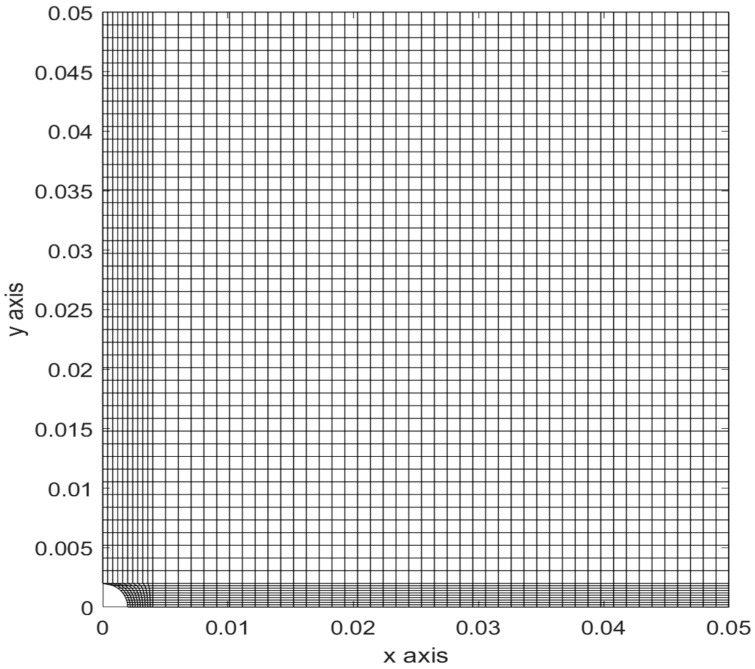
Finite element mesh of a quarter of a plate with a center hole.

**Figure 4:**
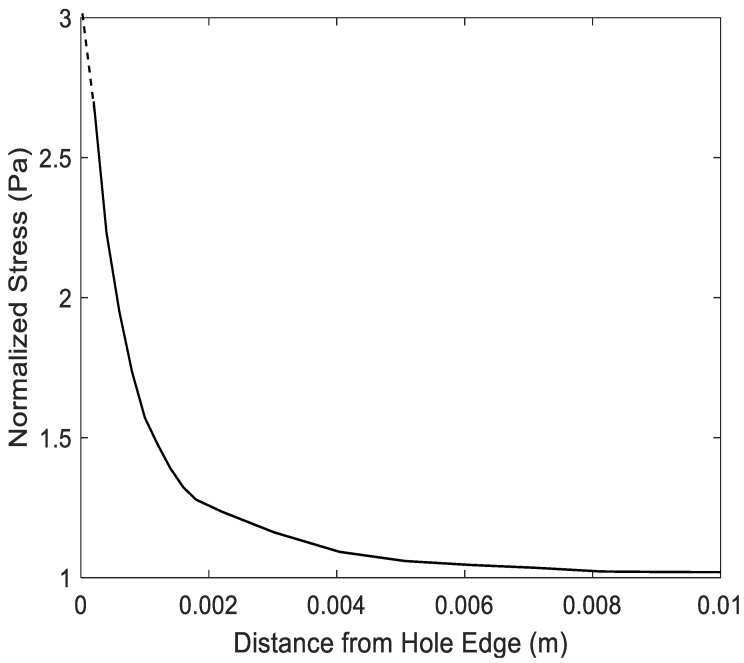
Finite element solution of y-normal stress along *x*-axis of a quarter of a plate with a center hole.

**Figure 5:**
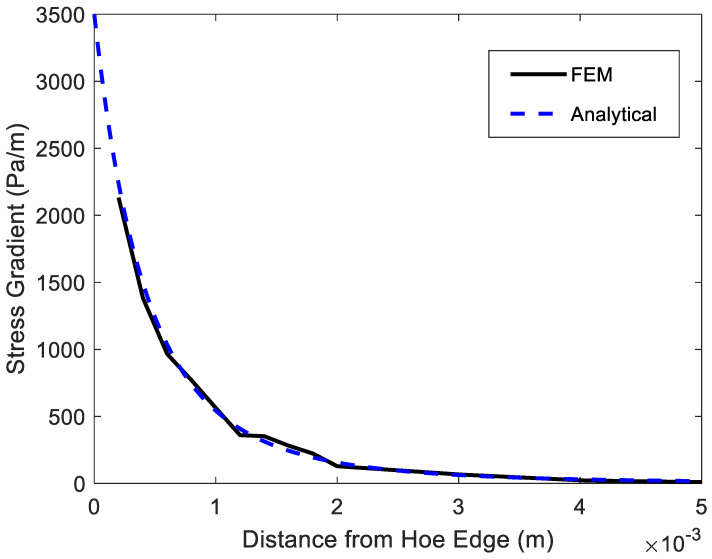
Stress gradient from the edge of a hole of an infinite plate.

Figure 3 shows a finite element mesh of a quarter symmetric plate with *a* = 2 mm and $\sigma_{o}=1$ Pa. The plate was 100 mm by 100 mm to imitate an infinite plate. The mesh had 3025 four-node quadrilateral elements. Both stress and stress gradient are plotted in Figure 4 and Figure 5 for the unit value of the applied stress. The stress concentration factor at the edge of the hole was 3 for an infinite plate subjected to uniaxial loading. Figure 4 shows the stress variation from the edge of the hole. Because stresses were computed at the center of every element, the plot needs to be extrapolated for the stress concentration factor at the edge of the hole. The broken line in Figure 5 shows that the stress concentration factor at the edge of the hole was 3.

Figure 5 shows the stress gradient plot from the edge of the hole. The analytical value was 3500 Pa/m at the edge of the hole. Again, the stress gradient was computed at the center of every element. If the finite element solution is extrapolated to the edge of the hole, the finite element solution of stress gradient agrees well with the analytical solution.

**Figure 6:**
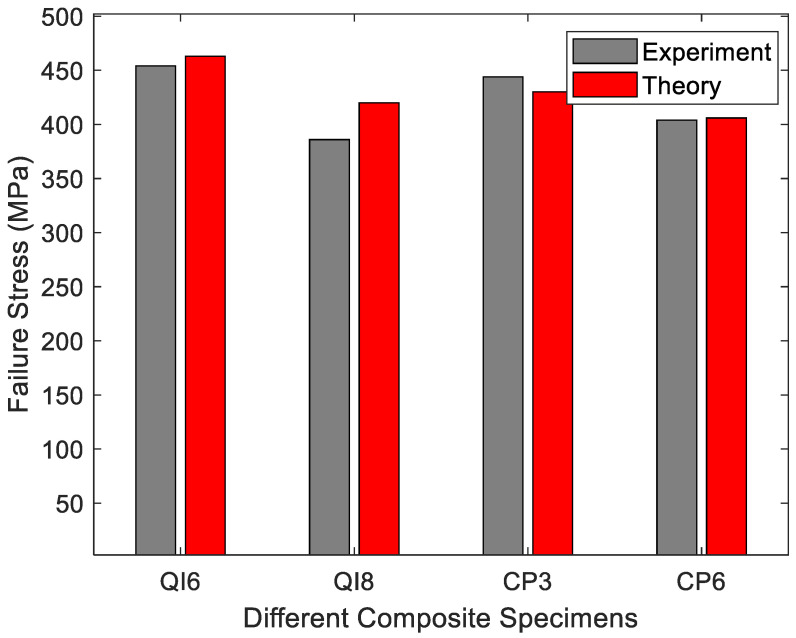
Comparison of failure stress between analytical and experimental results of laminated carbon fiber composite specimens with a hole at the center.

Once the finite element mesh was verified to provide an acceptable solution, similar meshes were used for analysis of laminated carbon fiber composites with a hole. Figure 6 compares the predicted failure stress to the experimentally measured stress. In the figure, ‘QI6’ denotes the QI specimen with a 6 mm hole, with similar notations for others. The stresses are the average applied stresses by the grip. For the experimental result, the average stress was computed by dividing the failure load at the grip by the cross-sectional area of each specimen. The finite element model used the displacement as applied loading to simulate the testing condition. Then, the total reaction force at the grip boundary was divided by the cross-sectional area of the specimen at the grip site to determine the applied failure stress. The predicted failure stresses agreed well with the experimental results for both CP and QI specimens.

One of the main advantages of the multiscale approach is to conduct parametric studies very efficiently to understand the effect of a selected parameter on the strength and stiffness of composite structural members. For example, the fiber volume fraction was varied for the CP composite specimen with a hole of 6 mm diameter. The parametric study showed that the failure strength varied along with the fiber volume fraction. The variation was quite linear. That is, as the fiber volume fraction increased by 5%, the failure strength increased nearly 5%.

Another parametric study was conducted for the lamination angle of the same CP composite. The 0° angle remained the same as it was along the loading direction. Instead, 90° layers were varied by ±2.5% or ±5%. The former case increased the failure stress by 3.6%, while the latter case increased it by 9.6%.

## 6. Conclusions

The new unified failure criterion is coupled with a multiscale failure analysis model in order to predict failure of the polymer composites containing any shape of a notch. The multiscale analysis links the micro-scale and macro-scale bidirectionally. The micro-scale is the fiber and matrix material level, while the macro-scale is the homogenized composite material level. Failure criteria are applied at the micro-scale level. In other words, micro-stresses/strains at the fiber and matrix materials are used for failure criteria. There are three failure modes at the micro-scale: fiber failure, matrix failure, and fiber-matrix interface failure. Furthermore, the unified failure criterion is applied to the micro-scale. The unified theory can be applied to any specimen regardless of whether it has a crack, cutout, or none of them.

The unified failure criterion has two parts. One part is the stress condition, and the other is the stress-gradient condition. In order to initiate failure, both conditions must be satisfied simultaneously. The stress condition is to determine critical locations whose effective stress exceeds the strength of the material. The stress-gradient condition checks the neighboring condition of the critical locations. If the stress gradient is too large, failure does not occur. The stress gradient condition can provide the potential failure path initiated from the critical location.

The unified failure criteria implemented in a multiscale approach are assessed using laminated carbon fiber composite specimens with a hole at the center to predict their failure stresses. The numerically predicted failure stresses are compared to experimental results. Both results agree very well. Even though two different laminates, such as CP and QI, are tested, the same failure strength values can be used at the microscale regardless of the lamination angles because the failure strength at the microscale is independent on the composite layup. This is not the case in the traditional macroscale analysis. Thus, the present multiscale approach associated with the new unified failure criterion can eliminate unnecessary tests to predict failure of various laminated composites with many different types and shapes of defects.

## Figures and Tables

**Table 1:** Material properties of fiber and matrix.

Fiber longitudinal modulus	220 GPa
Fiber transverse modulus	28 GPa
Fiber inplane shear modulus	30 GPa
Fiber inplane Poisson’s ratio	0.12
Fiber out-of-plane Poisson’s ratio	0.4
Matrix elastic modulus	3 GPa
Matrix Poisson’s ratio	0.3
Fiber failure strength	2.55 GPa
Matrix failure strength	20 MPa
Interface shear strength	80 MPa
Interface normal strength	54 MPa
Fiber volume fraction	0.5

## Data Availability

The data presented in this study may be available on request.

## References

[1] Hill R. (1950). Mathematical Theory of Plasticity.

[2] Tsai S.W., Schwartz R.T., Schwartz H.S. (1968). Strength theory of filamentary structures. Fundamental Aspects of Fiber Reinforced Composites.

[3] Tsai S.W., Wu E.M. (1971). A general theory of strength for anisotropic materials. J. Compos. Mater..

[4] Hashin Z., Rotem A. (1973). A fatigue failure criterion for fiber reinforced materials. J. Compos. Mater..

[5] Hashin Z. (1980). Failure criteria for unidirectional fiber composites. J. Appl. Mech..

[6] Sun C.T., Quinn B.J., Tao J., Oplinger D.W. (1996). Comparative Evaluation of Failure Analysis Methods for Composite Laminates.

[7] Hinton M.J., Kaddour A.S., Soden P.D. (2004). Failure Criteria in Fibre Reinforced Polymer Composites: The World-Wide Failure Exercise.

[8] Kwon Y.W., Darcy J. (2018). Failure criteria for fibrous composites based on multiscale modeling. Multiscale Multidiscip. Model. Exp. Des..

[9] Kwon Y.W., Darcy J. (2018). Further discussion on newly developed failure criteria for fibrous composites. Multiscale Multidiscip. Model. Exp. Des..

[10] Kwon Y.W., Panick C.J. (2020). Strain rate dependent failure criteria for fibrous composites using multiscale approach. Multiscale Multidiscip. Model. Exp. Des..

[11] Neuber H. (1958). Theory of Notch Stresses: Principles for Exact Calculation of Strength with Reference to Structural Form and Material.

[12] Peterson R.E., Sines G., Waisman J.L. (1959). Notch-sensitivity. Metal Fatigue.

[13] Npvozhilov V. (1969). On a necessary and sufficient condition for brittle strength. Prik. Mat. Mek..

[14] Whitney J.M., Nuismer R.J. (1974). Stress fracture criteria for laminated composites containing stress concentrations. J. Compos. Mater..

[15] Taylor D. (1999). Geometrical effects in fatigue: A unifying theoretical model. Int. J. Fatigue.

[16] Taylor D. (2008). The theory of critical distances. Eng. Fract. Mech..

[17] Sapora A., Torabi A.R., Etesam S., Cornetti P. (2018). Finite fracture mechanics crack initiation from a circular hole. Fatigue Fract. Eng. Mater. Struct..

[18] Braun M., Müller A.M., Milaković A.-S., Fricke W., Ehlers S. (2020). Requirements for stress gradient-based fatigue assessment of notched structures according to theory of critical distance. Fatigue Fract. Eng. Mater. Struct..

[19] Kwon Y.W. (2021). Revisiting failure of brittle materials. J. Press. Vessel. Technol..

[20] Kwon Y.W., Diaz-Colon C., DeFisher S. (2022). Failure criteria for brittle notched specimens. J. Press. Vessel. Technol..

[21] Kwon Y.W. (2015). Multiphysics and Multiscale Modeling: Techniques and Applications.

